# As if you were hiring a new employee: on pig veterinarians’ perceptions of professional roles and relationships in the context of smart sensing technologies in pig husbandry in the Netherlands and Germany

**DOI:** 10.1007/s10460-023-10450-6

**Published:** 2023-05-01

**Authors:** Mona F. Giersberg, Franck L. B. Meijboom

**Affiliations:** Animals in Science and Society, Department Population Health Sciences, Faculty of Veterinary Medicine, Utrecht University, Utrecht, Netherlands

**Keywords:** Pig production, Precision livestock farming, Society, Interview, Thematic analysis

## Abstract

Veterinarians are increasingly confronted with new technologies, such as Precision Livestock Farming (PLF), which allows for automated animal monitoring on commercial farms. At the same time, we lack information on how veterinarians, as stakeholders who may play a mediating role in the public debate on livestock farming, perceive the use and the impact of such technologies. This study explores the meaning veterinarians attribute to the application of PLF in the context of public concerns related to pig production. Semi-structured interviews were carried out with pig veterinarians located in the Netherlands and Germany. By using an inductive and semantic approach to reflexive thematic analysis, we developed four main themes from the interview data: (1) the advisory role of the veterinarian, which is characterized by a diverse scope, including advice on PLF, generally positive evaluations and financial dependencies; (2) the delineation of PLF technologies as supporting tools, which are seen as an addition to human animal care; (3) the relationship between veterinarian and farmer, which is context-related, ranging from taking sides with to distancing oneself from farmers; and (4) the distance between agriculture and society, in the context of which PLF has both a mitigating and reinforcing potential. The present findings indicate that veterinarians play an active role in the emerging field of PLF in livestock farming. They are aware of and reflect on competing interests of different groups in society and share positions with different stakeholders. However, the extent to which they are able to mediate between stakeholder groups in practice seems to be constrained by external factors, such as financial dependencies.

**Supplementary Information:**

The online version contains supplementary material available at 10.1007/s10460-023-10450-6.

## Introduction

Pig production in Europe is linked to many societal challenges. In a previous review of the literature we found that the public holds rather negative attitudes towards current pig husbandry conditions (Giersberg and Meijboom 2021). It appears that the direction of criticism is not uniform: the level of concern varies among subgroups of citizens and different aspects of pig production. This is contrasted by farmers’ more positive views on pig production, whereas the attitudes of other stakeholders, such as veterinarians and advisors, have rarely been explored (Giersberg and Meijboom 2021). In a multi-stakeholder comparison it has been shown that pig veterinarians share both the negative attitudes of citizens and the positive attitudes of farmers, depending on the particular aspect of pig husbandry under study (Bergstra et al. 2017).

It is often claimed that the introduction of smart sensing technologies may be the solution to various problems of current pig production, including societal concerns on animal welfare or environmental impacts (e.g. Vranken and Berckmans 2017; Tullo et al. 2019). However, to substantiate or reject this claim, further reflection is needed (Giersberg and Meijboom 2021). A first step would be to gather and analyze empirical data on the perceptions and attitudes of the key stakeholders involved regarding these technologies. Despite the large amount of scientific literature on the development and application of these sensor-based Precision Livestock Farming (PLF) tools (e.g. Gómez et al. 2021), little research has been done on how these technologies resonate with public attitudes towards pig production (Giersberg and Meijboom 2021). First results of focus group discussions show that consumers are hardly aware of the emergence of sensor technologies in livestock farming (Krampe et al. 2021). After having received a short explanation of the concept of PLF, participants indicate that they would expect these technologies to improve the health and welfare of the animals, the environment, transparency and the control of processes and stakeholders (Krampe et al. 2021). However, at the same time, consumers express their fears that PLF would lead to a robotization of livestock farming, that it may be prone to data manipulation, and that it may not be communicated properly to the public (Krampe et al. 2021). The few studies on farmers’ attitudes indicate that for them, the perceived practicality and usefulness of PLF technologies are decisive for evaluating their use more positively or negatively (Hartung et al. 2017; Lima et al. 2018; Giersberg and Meijboom 2021). Veterinarians can also be regarded as important stakeholders in this context: they may be confronted with PLF technologies during their work on the farms, they have obligations towards their farmer clients, but their profession also requires the consideration of broader public interests, such as animal welfare or public health (Meijboom 2018). However, empirical data on the attitudes of veterinarians towards the use of PLF tools in pig husbandry in Europe are not available (Giersberg and Meijboom 2021).

On the basis of the existing body of literature it is not possible to analyze whether PLF technologies may be a means to tackle or reinforce public concerns related to current pig production. We lack information on the attitudes of key actors in the field. Discussing the societal acceptability of PLF only within conceptual approaches may fail to embrace actual experiences and ideas of the people involved. Similar constraints may apply to quantitative and sometimes also qualitative investigations in which participants have to rate predetermined items, such as statements or pictures, or give answers to a set of very specific questions. These approaches may produce socially desirable responses within existing frames and stereotypes (Krystallis et al. 2009) instead of enabling profound understanding of the study participants’ reality. Therefore, the aim of the present study is to explore and grasp the meaning informants in their natural setting ascribe to the use of PLF technologies in pig production. As informants we selected veterinarians. Their practical work on the farms, their contact with the farmer, the animals and the technology makes them important stakeholders, yet they are largely underrepresented in empirical studies on attitudes towards current challenges of livestock production.

In line with the research aim stated above, we do not test a distinct a priori hypothesis. Nevertheless, we consider the following assumptions in our study design: (1) PLF technologies may not be widespread on pig farms, and (2) overall digital progress varies among countries and may influence people’s attitudes towards PLF. Despite the large body of research on PLF technologies, their actual uptake on common pig farms is uncertain. The adoption of such technologies remains a challenge and seems to be most advanced on larger, specialized dairy farms (Van Hertem et al. 2017; Groher et al. 2020). Therefore, in this study, we define PLF or smart sensing technologies in the broadest sense. PLF means the management of livestock by using the principles and technologies of process engineering (Wathes et al. 2008). It basically relies on sensors which monitor animals and related environmental variables with a varying degree of automation (Wathes et al. 2008). In this definition, we include both tools that are as simple as individual feeding stations, and more sophisticated technologies, such as computer vision approaches for automated animal behavior recognition. We further assume that the adoption of and the attitudes towards smart sensing technologies may be influenced by the overall digital progress of a given country. Therefore, we chose to talk to veterinarians in the Netherlands and in Germany. The legal regulations of the European Union apply to both countries, and the housing conditions of pigs are relatively comparable (Sørensen and Schrader 2019). However, the current level of digitalization differs substantially between the Netherlands and Germany. Across Europe, the Netherlands scores high for a set of digital progress indicators, particularly for embedding digital technologies into businesses’ activities (European Commission 2021a). In contrast, Germany scores lower for all indicators, and even ranks below the European average for the integration of digital technologies (European Commission 2021b).

In this article, we present and interpret veterinarians’ accounts of the application of PLF technologies in pig husbandry in the Netherlands and Germany. Based on these empirical findings, we discuss the potential role of PLF in addressing societal concerns related to current pig production.

## Methodology

### Context of the study and reporting

As the aim was to explore and understand the meaning pig veterinarians ascribe to the use of smart sensing technologies in pig husbandry, we determined an approach within the values and assumptions of a qualitative paradigm to be most appropriate (Creswell 2014). We further decided to work within a critical realist epistemology assuming a simple, more unidirectional relationship between the research participants’ meaning and experience and their language (Braun and Clarke 2006). However, it is important to notice that critical realism acknowledges an inherent subjectivity, such as the researcher’s interpretations, in the generation of knowledge, which represents a certain overlap with a contextualist position (Madill et al. 2000). Therefore, all data collection and analyses were carried out by the first author (MFG). The present study is reported according to the criteria mentioned in the COREQ guideline (Tong et al. 2007), which are detailed in Online Resource 1.

The first author, MFG, is female, trained as a veterinarian, and holds a post-graduate degree in veterinary science. She is employed as researcher animal welfare/sustainable animal stewardship at Utrecht University, the Netherlands. MFG is a native German speaker with high proficiency in English and Dutch.

### Participants

Based on the results of our previous literature review (Giersberg and Meijboom 2021), we were interested in gaining insight into the perceptions and opinions of pig veterinarians regarding the use of smart sensing technologies on farms. Inclusion criteria were that potential participants had to be pig veterinarians (defined as veterinarians mainly or exclusively engaged in disease prevention and cure of pigs), worked in a veterinary practice, and were located in the Netherlands or in Germany. The authors asked academic colleagues from more applied fields of veterinary science to suggest pig veterinarians they knew. In this way, a list of 25 candidates was created. Persons from this list were invited via e-mail to take part in an interview in the context of the present study. Ten pig veterinarians did not respond to the e-mail invitation, two declined participation because of retirement and one declined participation because of time constraints. In total, 12 pig veterinarians agreed to participate. MFG did not know the participants personally or had worked with them prior to the study. The participants, in turn, knew that MFG is a researcher interested in animal welfare and the socio-ethical aspects of this topic. During the conversation, they were also told that MFG was trained as a veterinarian in Germany but has been working ever since in research.

### Data collection

Data were collected during semi-structured interviews. This method allows the participants to phrase their opinions in their own words and enables the interviewer to use follow-up questions flexibly based on the participants’ responses (Kallio et al. 2016), which fits our aim to gain a detailed view of informants in their natural setting on the topic under research. The interview guide was developed according to Castillo-Montoya’s interview protocol refinement framework (Castillo-Montoya 2016). Based on our overall research question, we developed broad sub-questions and aligned them with the interview questions in an interview protocol matrix (Online Resource 2). In order to avoid too much framing by the researchers, the participants were not provided with a detailed definition of the concept of PLF. They were only given the following broad explanation to introduce the topic of the interview: “PLF is the management of livestock by means of the principles and technologies of process engineering. It relies on automatic monitoring of animals and related environmental processes by various smart sensing technologies. The degree of integration and automation of these technologies varies.” In addition, the interview guide included an ‘incident’ which means that participants were asked to describe a past event where they came into contact with PLF. This allows us to interpret which technologies, sensors or specific tools the veterinarians view as belonging to PLF.

The interview was practised with colleagues from our research group, and they were asked to give feedback on the interview guide. However, the sample of potential participants was too small to recruit some of them for piloting. The interview guide was prepared in English and translated by MFG into Dutch and German in order to hold the conversations with the participants in their native language.

The interviews were carried out between November 2020 and March 2021. Because of travel restrictions due to the Covid-19 pandemic, all interviews were held online via MS Teams. The participants joined either from home or from their office. Of the 12 pig veterinarians, seven participants (one female, six males) with a work experience of 3–35 years were from the Netherlands. The remaining five participants (one female, four males) were from Germany and had a work experience of 6–31 years. During data collection we learned that several participants were working in both the Netherlands and Germany or had close collaborations in the other country. Therefore, we rejected our previous assumption that participants’ views on PLF may be influenced by the level of digitalization of the country they were based in, and treated all participants as one sample.

Prior to the interviews, the participants received a consent form which they signed and returned via e-mail. With their signature they agreed to participate in the study, to the use of anonymized written extracts from the interviews for publication, and to being or being not video- and audio recorded for detailed transcription and analysis of their responses. They were also informed that they could withdraw from the study at any time without giving reasons. The participants further received a data management plan explaining how their data were handled according to the effective General Data Protection Regulation. In the beginning of each interview, the consent form was reviewed together with the participant, and it was asked whether they still agreed to video- and audio recording the conversation or not. All interviewees, except for one, gave permission to being video- and audio recorded using the recording function of MS Teams. The recordings were transcribed by MFG. In the one case in which consent for recording was not given, notes were taken during the interview almost verbatim.

### Data analysis

Data were managed and analyzed using NVivo 12 pro (QSR International). The transcripts were handled in their original language (Dutch, German). Starting with the generation of initial codes, all steps of the analysis were carried out in English. Participants’ quotes that serve as illustrations in this paper were translated by the authors.

In line with the qualitative paradigm, our aim was to gain insight into the views of individuals belonging to a certain group (pig veterinarians) regarding PLF technologies, not to test a hypothesis or to provide a representative account of the topic. The current body of literature (see also Giersberg and Meijboom 2021) was insufficient to develop themes and a coding frame prior to data analysis. Therefore, we used a rather inductive and semantic form of reflexive thematic analysis (TA) to analyze the interview data (Braun and Clarke 2006, 2020). This data-driven approach to TA assumes that themes can be developed by finding shared patterns of meaning within the participants’ responses. ‘Meaning’ at a semantic level is considered to be explicit within the language of the participants. Based on these assumptions, relevant data across the entire data set were collated to initial codes. These codes were refined and merged where appropriate. In the following, codes were combined to create potential themes. By re-reading all coded data extracts, candidate themes were reviewed and refined resulting in final themes. The interpretation of final themes was carried out by means of further re-reading, reference to literature, and consultation between the authors.

## Results

The analysis resulted in the development of four main themes reflecting the meaning pig veterinarians ascribed to their own role, PLF technologies as such, their relationship with other stakeholders, and relationships among stakeholders. These themes were named: (1) the veterinarian as advisor, (2) PLF technologies as supporting tools, (3) the relationship between veterinarian and farmer, and (4) the distance between agriculture and society.

### The veterinarian as advisor

During the interviews, the veterinarians recurrently considered their own role, which all of them framed as advisory. Here, we focus on two ways in which the participants reflected on this advisory role: (a) by outlining the scope of their advisory activities, and (b) by evaluating their role as advisor.

#### (A) scope of advisory activities

Consistent with the participants’ background in veterinary medicine, advisory activities were in general focused on pig health. In this context, particular importance was attached to the prevention of health problems. One veterinarian explicitly distinguished these activities from curative tasks:

*“So, just really the advice. Clinically I don’t do that much, I don’t do sick pigs, yes, very occasionally I see a sick pig, but* […] *I do that actually sporadically.”* (DA2).

Another participant went a step further, regarding curative tasks not only as not belonging to, but also as contrary to advisory activities:

*“I’m actually no longer involved in treating disease problems in pigs. Well honestly, if I have to do that, I consider it as a loss. Then I did something wrong in the trajectory prior to that point.”* (DA3).

Furthermore, the own advisory activities were separated from the making of decisions, which was considered the task of the farmer:

*“Because here I have an amazing position on the pig farms, at the table where decisions are made, I don’t have to decide myself, but I can advise.”* (DA1).

Some participants narrowed the scope of their role to that of an “advisor on animal health” by distinguishing their activities from those of other advisors on the farm, such as “advisors on feed”. In contrast, other veterinarians regarded advice on feed, genetics, barn structure and barn climate as part of their responsibilities. The practical form of advisory activities ranged from very specific suggestions, such as on how to perform certain procedures on the animals, to support in data interpretation, to a more holistic account of advice, which one participant described by using the following metaphor:

*“So, this role has become increasingly important to me, so just circling above the farm like a helicopter* […].*”* (DA1).

A similar variety of accounts can be observed when having a closer look at the roles the veterinarians attributed to PLF technologies within their advisory activities. These are on a continuum from not playing a role at all to being a core subject of veterinary advice. At the one end of the continuum is the expression that PLF technologies do not fall within the scope of veterinary advisory activities because the advised farms “don’t have anything like that”, or, if they had, data were not or only selectively shared with the veterinarian. At the other end of the continuum, participants stated that it is part of their regular tasks to advise on PLF technologies and the resulting data output. Several interviewees (n = 5) even stated that they are actively involved in the development, testing and provision of PLF tools for pig farmers:

“[…] *and we even founded a software company ourselves, where we also have our own software* […] *and are already for a few years now almost directly connected to the farmer.”* (TA4).

*“I even have a* […] *mobile phone with a thermal imaging camera, so I can use this phone and for example* […] *see if there are any draft holes in the barn.”* (DA2).

*“I think we are very proactive with that* [PLF], *also because we are already starting to work with the equipment in the test phase and then we do beta testing on a number of farms and then, eventually, we want to use it commercially.”* (DA6).

One veterinarian also stated that in the future, veterinary education should focus more on advisory activities regarding PLF technologies:

*“And what I miss is that education is not focused on this. If we as veterinarians want to continue to do this well, we must have knowledge and that’s what I miss with the students. That’s not because of the students themselves, it’s because of the information they are given.”* (DA1).

#### (B) evaluation of the advisory role

In addition to describing and delineating their advisory activities, the pig veterinarians further reflected on their role by evaluating it. Some participants were explicitly positive about their role. Positive associations were expressed as “having fun working as a veterinarian” and “enjoying work”, and typically arose when advisory activities were discussed. One veterinarian put it as follows:

*“[I] am mostly working with people, and that’s what really makes my job so much fun. I’m really happy that I can go out to the farms every day.”* (DA3).

Another participant expressed their positive view in terms of career prospects:

*“I do expect to continue to work in the pig sector for the rest of my career, that’s definitely my goal.”* (DA2).

However, participants, including the ones who evaluated their role positively, also mentioned fields of tension. The main point mentioned was the challenge to consider the, sometimes conflicting, interests of different stakeholders during the advisory activities. In this process, the animals were naturally regarded as stakeholders with their own interests:

*“The interest of the animal may sometimes be that I treat it with antibiotics, but society doesn’t want that.”* (DA3).

The main problem was not seen in dealing with these interests and tensions per se:

*“I’m somewhere in between* [the competing interests], *I can function very well because I’m academically trained, I’m supposed to be an autonomously thinking professional* […].*”* (DA3).

It was rather the financial dependency on the farmer as client that was perceived to sometimes impede the advisory activities of the veterinarian:

*“[…] but I have an enormous dependency on the pig farmer, because he pays my bill.”* (DA3).

*“We are supposed to ensure animal health and of course we have to liquidate the whole thing, which means that the farmer pays us for a service that […] costs him money, which may not help him much at first sight […], and that is of course very difficult, because the understanding is not always there for certain things.”* (TA3).

*“So, we always need to have this sensitivity towards the client but also to be an advocate for the animals.”* (TA5).

In summary, the veterinarians were unanimous in having an advisory role. However, what this role entails in detail seems to be diverse. In some cases the scope was restricted to more traditional advice on animal health, whereas in other cases it included the active provision of PLF tools. When the participants talked about their role in an evaluative way, the most prominent aspects mentioned were positive connotations and limitations of advisory activities due to the financial dependency on the farmers as clients.

### PLF technologies as supporting tools

The second theme that was developed by analyzing the interviews is the participants’ conceptualization of PLF technologies as supporting tools. This view on PLF was consistent across all interviewees. In analogy to the accounts on the advisory role of the veterinarian, this theme can be structured into (a) statements that (indirectly) discriminate the supporting role against other possible roles of PLF and (b) evaluative talk on the presumed supporting role.

#### (A) scope of the supporting role of PLF technologies

Conceptualizing PLF technologies as tools that are “supportive” and “in addition” to something else implies a distinction from autonomous, independently operating systems. Interestingly, the veterinarians did not mention this delimitation explicitly; they rather used the implications of the concept of the supporting tool as a self-evident part of their elaborations on PLF. PLF technologies were mainly seen to support human observation of and care for the animals.

*“We have many good examples of where this additional technology is used meaningfully in combination with all the other criteria, including live animal observations on site, in the barn.”* (TA2).

*“It has to be supportive, yes, and at the end of the day, it is people who handle the animals* […].*”* (DA1).

*“Sensors are very important, but in addition, it is of course up to the farmer to see whether an animal is feeling well or not.”* (DA2).

Some interviewees expressed the same line of thought in a more restrictive way, i.e. that PLF technologies cannot replace human observation and care.

*“*[…] *without staff it just doesn’t work, so we can come up with many technological things, yes, but if you don’t observe the animal to a certain extent, or don’t look after the animal somehow at certain stages of production*, […] *then it just doesn’t work.”* (TA3).

*“Data, of course, can never capture the image of the whole animal, so there have to be people who say*, […] *I’ve been here and according to me this is not correct, or this situation is excellent.”* (DA5).

*“*[…] *that* [farmer’s expertise] *is indeed partly irreplaceable by a technological device, in my opinion.”* (DA6).

The veterinarians mentioned a number of ways in which the supporting qualities of PLF technologies could be applied in practice. It seems apparent that these technologies always came into play when certain information on the animals, the environment or on processes on the farm was supposed to be collected. This information was characterized by being additional (i.e. the farm could in principle be run without this information, although probably less efficiently) and by exceeding the measuring and observing capacities of human caretakers. One example is the use of automatic weighing scales to monitor the pigs’ individual body weights on a daily basis:

*“In new buildings, some already install gates with automatic weighing scales and cameras, so that they can check the body weights daily, so that they can manage the feed uptakes much better […].”* (TA1).

On the one hand, this illustrates the additional character of the information obtained by the PLF system, as farmers are obviously able to rear pigs without being aware of their daily body weights. On the other hand, it would exceed the caretakers’ capacity to weigh all pigs of a farm individually on manual scales each day to gain this information. Other examples mentioned were sensors measuring the ambient climatic conditions in a barn, a system monitoring the coughing of pigs as an indicator for respiratory infections, and water meters measuring water consumption per group of pigs. However, several participants challenged this account of the scope of PLF technologies. According to them, PLF systems should take over routine work of the farmer.

*“I would say it has to* […] *save working time, it has to simplify things, of course, simplify processes, that’s clear* […] *but as said, it must provide a workload reduction.”* (TA3).

Although this view is not focused on additional information, it is still in line with the supporting role of PLF: these technologies should only reduce the workload and save time, they are not seen to replace human evaluation and intervention.

#### (B) evaluation of the technological support

During the interviews, the veterinarians also rated the delineated supporting role of PLF technologies. Several participants (n = 8) evaluated this role positively and identified a “great future potential” for these technologies. Again, the characteristics of “additionality” and “exceeding human measuring capacities” of the information obtained by PLF systems were addressed:

*“I think there are these systems that assess situations in the barn, climate, weight of the animals and so on, I think that makes absolute sense and has a great future.”* (TA4).

*“On the animal itself I think*, […] *there are good things to measure and if you also include a number of environmental factors, you can improve by comparing all your data*, […] *I think that has added value.”* (DA5).

Another veterinarian expressed even more explicitly that PLF technologies are seen positively when supporting human animal observation and care:

*“*[…] *continuing to look at the pigs, continuing to look at the piglets and also providing them with individual care, then yes, I would be happy with the developments on a level that you measure what the growth is daily or that you measure whether there is more than average coughing.”* (DA3).

Furthermore, critical views were shared, also by those participants who were generally positive about the supporting role of PLF technologies. One issue were financial aspects, i.e. the veterinarians regarded the technologies as absolutely too expensive or too expensive in relation to their (economic) benefits for the farmers. Another point of criticism the participants expressed can be linked to the scope of PLF outlined above. First, it may be too difficult to operate these systems that provide additional information exceeding human observation capacities:

*“I see a crucial point in the ease of use of the technology, because they* [the farmers] *have extreme problems finding skilled personnel here, and it’s really hard to find someone who can operate technology like this.”* (TA4).

Second, these systems may provide too many data:

*“If there are even more data, we are swamped with data and you can’t see the forest for the trees anymore.”* (DA4).

Which, finally, may be difficult to interpret:

*“The subsequent interpretation of the data and the processing of the data, and linking management changes or whatever else to it, is difficult. Most pig farmers are not yet ready to deal with such a huge amount of data and to consequently focus on the right things.”* (DA1).

However, as PLF technologies were seen as novel and supporting humans by exceeding their capacities, their application would require more than knowledge in operation and data interpretation. This was described as a certain level of trust on the part of the farmer:

*“With that technology it is…as if you were hiring a new employee, because you have to learn to trust him that he…can do it…and a farmer has to dare to let go of that.”* (DA6).

Criticism was also voiced when it was presumed that PLF technologies should take over routine work and save time. In this context it was mentioned that technological support would not have added value for smaller farms:

*“On small and medium-sized farms*, [human] *monitoring is still such that it* [PLF] *doesn’t add much value.”* (DA5).

PLF may even pose a risk of further upscaling of pig farms:

*“I am afraid that many technologies will be used to be able to take care of even more pigs with fewer people* […]*”* (DA3).

In summary, the veterinarians framed the actual scope of PLF in different ways, but they all agreed on the supporting role of these technologies. Two views were prominent: first that PLF would support farmers in gaining additional information that would exceed human measuring capacities, and second that PLF should support farmers by taking over routine work and saving time. In general, this delineated role of PLF was rated positively, with having a great potential. However, critical views were also expressed, mainly relating to feasibility and necessity of PLF.

### The relationship between veterinarian and farmer

The themes presented above, the advisory role of the veterinarian and the supporting role of PLF technologies, naturally entail representations of the relationship between veterinarian and farmer. A field of tension regarding one’s own role, for instance, can only be recognized in relation to others. However, this third theme covers aspects beyond that. When reflecting on certain topics, the veterinarians were “taking sides” with or showing some kind of compassion for the farmers. One of those topics was the compliance with regulatory or legal frameworks for pig production. These regulations were generally considered to be “challenging” or “overburdening” for the farmer:

*“The development here is that pig farming is of course becoming more and more difficult in terms of the general conditions and also the legal requirements.”* (TA4).

*“So, more and more obligations keep coming, but he* [farmer] *also has to feed his sows, you know, there’s so much coming over him* […]. *In the end I do have a bond with the farmers, they are my customers, and it is close to my heart if they are not doing well.”* (DA4).

Economic pressure was seen as another difficulty the farmers have to deal with:

*“The profits are incredibly small in pig farming, that is their fate, making it only possible to think in terms of volume, of maintaining a family income, rather than in terms of quality.”* (DA3).

Together, these challenges lead to uncertainty:

*“The biggest trouble is that they* [farmers] *themselves do no longer know where pig production is heading to.”* (TA1).

*“I see uncertainty in economy, I see uncertainty in regulations, which means that farmers are not moving ahead with their businesses.”* (DA1).

Finally, the sympathy of the veterinarians with the farmers was expressed by stating that – despite the unfavorable conditions – “most farmers do their best”, particularly when compared to pig production in other countries:

“[…] *a pig lies in a pen and is well cared for in 99% of cases by the farmer* […]” (DA4).

*“That is really my opinion, we are well positioned in Germany compared to many other countries.”* (TA2).

*“*[…] *that there really are differences between how pigs are kept here* [the Netherlands] *and how they are kept in other countries.”* (DA6).

However, at the same time, veterinarians were distancing themselves from farmers, mainly when discussing subjects related to animal welfare and management. One participant summarized it as follows:

*“Because the agricultural sector is developing an animal welfare standard where I ask myself whether that is animal welfare at all.”* (TA2).

In those statements, in which the veterinarians distanced themselves from farmers, they often referred to their advisory role:

*“My goal on almost all farms is: guys, this is the pig, that’s how it is made, you have to deal with it and adapt your system to the pig the way it is. This prevents a lot of problems.”* (DA3).

*“Those* [farmers] *who have no interest* [in innovation], *some of them are really like that, they can’t be taught”* (TA1).

Between these two extreme positions – taking sides with farmers and distancing themselves from farmers – the veterinarians also reflected in a more neutral way on their relationship with farmers. When the participants for instance brought up topics such as the reduction of pig production or the specialization of the sector, they neither took sides with the farmers nor distanced themselves from them. Therefore, the relationship between veterinarians and farmers can be characterized as diverse and multilayered, ranging from compassion to distance on the veterinarians’ side. Which concrete position was taken from this continuum seemed to be more related to the topic under discussion than to the individual veterinarian.

### The distance between agriculture and society

This final theme concerns the relationship veterinarians noticed between other stakeholders, i.e. between farmers or the agricultural sector and the wider society. The distance between agriculture and citizens in the Netherlands and Germany was mentioned by all participants and brought up recurrently during the interviews. The veterinarians’ statements can be grouped into aspects that were seen as a result of the present distance, aspects that may have caused and are likely to further increase the distance, and aspects that may mitigate it. A lack of knowledge about pig husbandry within the broader society was regarded as one of the results of the current distance:

*“They* [citizens] *have no idea how pigs are kept here.”* (TA1).

This lack of knowledge may lead to misconceptions, which on the one hand could be dystopic:

*“A view is often expressed of lots of pigs packed tightly together in dark pens with lots of shit.”* (DA3).

*“So there are a lot of prejudices about animals in the sector, they are always miserable and* […] *they are treated badly, there are antibiotics in the meat and all that kind of stuff you hear.”* (DA6).

On the other hand, misconceptions could be based on a utopic, traditional image of agriculture:

*“Everyone imagines that they* [pigs] *roam around on a green meadow* […].*”* (TA2).

*“*[…] *and when I take the perspective of a consumer, then I think yes, I would also rather have a pig that just came out of the woods* […].*”* (DA4).

According to the veterinarians, both types of misconception may result in a lack of societal acceptance of pig production under the current circumstances:

*“Yes, there is a lack of acceptance on the part of society of the, I would say, currently common husbandry conditions.”* (TA2).

*“*[…] *and, in particular, I think that farrowing crates and the housing of fattening pigs are perceived as problematic.”* (DA7).

Several veterinarians argued that with the current distance between agriculture and society, the use of PLF technologies may enforce misconceptions and the lack of acceptance of pig production:

*“They* [society] *might look at technology as oh, that’s even more looking at the animal in a mechanic or economic way, and that’s exactly what they don’t wan*t […].*”* (DA6).

*“If you are looking at it from society’s point of view, you are going to industrialize it* [pig production] *even more. It becomes very process oriented, it becomes like a car factory* […].*”* (DA4).

The interviewees saw the reasons for the observed distance and the lack of acceptance partly on the side of the agricultural sector. Some veterinarians reported that the sector “failed to create a positive image”, in which they included “the absence of appropriate marketing showing technological progress, as it is common in other economic sectors.” For others, the problem was that the agricultural sector failed to create a realistic image. This was linked to a lack of transparency of pig production, which takes place in “closed red brick buildings”. Other reasons for increasing the distance were attributed to the society, in which citizens “do not really want to know” or “are not interested in pig production”. A number of participants expanded this by stating that society was also not interested in technological detail of PLF tools, and that “the average citizen is not ready to hear such a story”. This view was relativized by some veterinarians who indicated the plurality of society in this context:

*“THE society does not exist.”* (TA3).

*“It seems to be the case that a small group* [of society] *has slightly different ideas than the large group.”* (DA1).

There may be groups within society that are not interested to get to know more about pig production because they either do not care or they do not accept pig production under any circumstances. In between these positions, there may be groups that are interested at least in some aspects of pig production. This may be a starting point to mitigate the distance between agriculture and society:

*“When it comes to animal welfare, I am sure that this is very appealing to society.”* (DA1).

One option for the agricultural sector to address this may be to “keep pigs more naturally”:

*“The animals are kept more outside again, they are more seen by people, that is definitely positive, I think, so that this contact is re-established.”* (TA5).

In this context, the use of PLF technologies was mentioned again, which could, by improving animal welfare, increase societal acceptance of pig production, and thus mitigate the distance between agriculture and society.

*“If* [PLF] *systems are developed that allow pigs to engage in natural behavior*, […] *then I think we can get people on board.”* (DA3).

Another option mentioned to decrease the distance was “to convince through facts”:

*“*[…] *hard facts, so at the end of the day you have to say, by the use of technology we have less cannibalism, we have less use of antibiotics and so on, and they* [society] *wouldn’t get past that, so you have to provide robust facts, I think.”* (TA4).

*“But if you then discuss with people and explain about waiting times and that we are trying hard to do our best*, […] *then people are getting interested, interested in learning more about this and then there is understanding for this.”* (DA6).

In summary, the veterinarians stated a distance between the agricultural sector and society, for which they held both parties accountable. This distance was not perceived as neutral, but was associated with misconceptions, which ultimately led to a lack of acceptance of current pig production by society. In this context, PLF technologies were considered both as means that may increase the observed distance and as means that may mitigate it. The interest of (part of) society in animal welfare was seen as a crucial starting point for the agricultural sector to tackle the distance, and thus the lack of acceptance of pig production.

## Discussion

There is a lack of information on the meaning veterinarians attribute to the use of PLF technologies in the light of current public concerns related to pig production in Europe. The aim of this study is to close this empirical gap. This is the first step for further socio-ethical analyses of PLF technologies as part of responsible research and innovation.

During the analysis of the semi-structured interviews with pig veterinarians we developed four main themes: (1) the advisory role of the veterinarian, which is characterized by a diverse scope, generally positive evaluations and financial dependencies; (2) the delineation of PLF technologies as supporting tools, which entails both positive and critical connotations; (3) the relationship between veterinarian and farmer, which is context-related, ranging from taking sides with to distancing oneself from farmers; and (4) the distance between agriculture and society, in the context of which PLF has both a mitigating and reinforcing potential.

It should be noted that these results are based on a reflexive thematic analysis (TA) of the empirical data. Within reflexive TA, themes are regarded as patterns of shared meaning across the data set. They are the final output of a process of data coding and repetitive generation and refinement of candidate themes (Braun and Clarke 2020). In contrast to other forms of TA, such as coding reliability TA and codebook TA, reflexive TA fully acknowledges the assumptions and values of a qualitative paradigm (Braun and Clarke 2020). As mentioned before, the analyst’s subjectivity is seen as a skill and resource for generating knowledge (Braun and Clarke 2020). Consequently, positivist quality criteria, such as inter-coder reliability, do not apply to the approach we chose for the present study.

### Inner reflection: the role of the veterinarian

It is not surprising that the veterinarians recurrently described and evaluated their own professional role during the interviews. There are currently no definitions or broadly accepted frameworks of veterinary professionalism (Mossop 2012). In addition, societal influences that alter the role of the veterinarian lead to a continuous transformation of the profession’s identity (Armitage-Chan et al. 2016). This requires a constant adaptation of the veterinarian’s skills and self-reflection on their role. If the changing professional demands exceed the adaptative capacity of the individual veterinarian, this can result in identity confusion and incoherence (Armitage-Chan 2019). The present account of the veterinarian as advisor is coherent within individual veterinarian’s responses and across the group of participants. Over the last decades the requirements of the transforming livestock sector have shifted from individual to collective health care and from curative to preventive strategies (Ruston et al. 2016; Meijboom 2018). By delineating their advisory role explicitly from curative tasks, and even regarding such tasks as failure, the participants demonstrate a high level of adaptation to the altered demands. It is interesting that even within the same veterinary field (porcine medicine), the scope of the advisory activities is diverse. Some participants focused on being ‘the health advisor’ among different advisors on a farm, whereas others claimed a much broader spectrum of advisory activities, including the use of PLF technologies. Developing a software company to manage client data or using a thermal imaging camera to connect climate data with disease prevention are excellent examples of how veterinarians enhance their services to strengthen their position in the market place (Ruston et al. 2016). However, the veterinarians also delineated their advisory activities from the making of decisions. Taking the position of the ‘neutral’ advisor can be interpreted as another self-protecting strategy in dealing with conflicts among different stakeholder interests (Owens 2015). This strategy is similar to the “rabbit scenario” described by Meijboom (2018): in the light of an overwhelming plurality of conflicting views, the veterinarian freezes like a threatened rabbit. However, in contrast to the rabbit, the veterinarians in the present study do not seem to be paralyzed. They voice their opinion, but draw back on “only giving advice”.

Adopting this strategy is not a sign that veterinarians are unable or unwilling to embrace the normative dimensions of their work. Our results show that veterinarians are indeed weighing and reflecting on conflicting interests. However, they face an imbalance between the relatively equal demands of the main stakeholders (society, farmers, animals) and the exclusive financial dependency on one stakeholder (farmer). In practice, this may impede veterinarians to act as fully independent professionals. This conflict between external obstacles (here: one-sided financial dependency) and personal convictions was identified as the typical moral challenge of German farm animal veterinarians by Dürnberger (2020a). Despite those tensions, most participants evaluated their role as pig veterinarian explicitly positive, which is in line with previous results by Dürnberger (2020b). In a survey, Dürnberger (2020b) found that German farm animal veterinarians liked their job in part because of the contact with the farmers, an aspect that was also mentioned during our interviews.

### Outer reflection: the role of smart sensor technologies

Whether the veterinarians regarded PLF as a core subject of their advisory activities, or whether it played no role in their daily work, they all framed it as supporting tool. In this way, the pig veterinarians separated PLF from fully autonomously operating technologies, which are for instance emerging in crop farming (Lowenberg-DeBoer et al. 2021). They specified their accounts even further by self-evidently implying a prominent position of human animal care in their conceptions of PLF technologies. PLF tools were seen to either collect additional information that exceeds human measuring capacities or to take over routine work, but not to replace human care. This may be explained by the notion that human animal care, in whichever form, is the core concept of veterinary medicine (Donald 2019). The veterinarians may have internalized this concept in a way that they can envisage a transformation of human animal care (e.g. by technological support), but not its elimination from animal husbandry. Another possible explanation for regarding the human as essential factor in animal care may be that currently applied PLF technologies lack the level of technical integration and sophistication to take over complex caring tasks (Tzanidakis et al. 2021).

Given that PLF technologies were seen as supporting human animal observation and care, it is not surprising that most veterinarians evaluated them positively. Indeed, many potential welfare threats of PLF, such as less quality time spent with the animals or the loss of the farmer’s own skills to detect problems, seem to arise from a deterioration of human care for animals (Tuyttens et al. 2022). In line with Tuyttens et al. (2022), concerns were expressed that technologies may be used to upscale pig farms without upscaling human workforce. However, when sharing critical views, the veterinarians mainly focused on aspects of feasibility and necessity of PLF technologies. Possible disadvantages of PLF, such as cost-efficiency, the need for expert knowledge and skills to operate the system and to interpret data, the lack of trust in technology, and the uncertainty of the actual value of the additional information, have also been reported previously (Hartung et al. 2017; Rojo-Gimeno et al. 2019; Schillings et al. 2021). The metaphor of the “new employee” used by one of the participants nicely captures the veterinarians’ account of PLF: the new employee cannot replace the other employees, the company must not over- or under-rely on them but trust them, and their commitments need to have real value for the company.

### Reflection on relations: the veterinarian and the farmer

During the interviews, the veterinarians recurrently talked about their professional relationship with farmers. These expressions about relationships seem to be independent of the particular role and evaluation that the participants attribute to the use of PLF technologies in pig husbandry. The veterinarians’ relationship with their farmer clients can be characterized as multilayered and context-related. Compassion for farmers was particularly shown regarding the ‘uncertain’ conditions under which they produce: regulatory or legal frameworks and economic pressure were considered as main challenges. However, when it comes to aspects of animal welfare, veterinarians seem to have higher standards than farmers. This is in line with results obtained by Bergstra et al. (2017) who found that veterinarians expressed more negative attitudes compared to conventional pig farmers towards animal-related aspects of current pig husbandry, such as pain management or tail docking. Similar to our present findings, veterinarians showed also sympathy with farmers, for instance regarding the aspect of securing a sufficient income or the mental burden of livestock farming (Bergstra et al. 2017). This context-dependent relationship between veterinarians and farmers can be construed as an alternative version of Meijboom’s (2018) “chameleon scenario”. In the original scenario, veterinarians adjust to the values and interests of the one stakeholder they are economically dependent on: the farmer. In the present study we see that veterinarians can act as a real chameleon: adapting to those stakeholders whose interests are closest to their own views in a given context. As illustrated in the results section, veterinarians represent for instance the interests of the pig in questions of animal welfare. However, this representation of interests is not absolute, it obviously entails the possibility for the veterinarian to withdraw on their advisory role as discussed above.

### Reflection on others’ relations: agriculture and society

The veterinarians reflected in detail on the distance they noticed between the agricultural sector and society. They also reasoned how this distance contributed to the lack of acceptance of current pig husbandry conditions and even provided ideas on how agriculture could reconnect with society. What is interesting here is that the participants did not delineate their own role in these elaborations. It may therefore be best characterized as the role of an ‘eyewitness’. An eyewitness may understand and talk about complex relationships and situations, but does not participate in the events. In the role of eyewitness, the veterinarian cannot be expected to proactively take on a mitigating or bridging role between agriculture and society as suggested previously (Giersberg and Meijboom 2021). It can be speculated that the reasons for not taking a more active stance are again linked to the one-sided financial dependency of the veterinarians.

When the veterinarians talked about PLF from a citizen’s perspective, they expressed concerns that these technologies could be perceived as means to further industrialize pig production. This indicates that for citizens, in contrast to the veterinarians, human animal care may not be implicit in the concept of PLF. As citizens may value human care for farm animals (Boogaard et al. 2011), such technologies may increase societal concerns regarding pig production. Indeed, citizens expressed the fear that PLF would facilitate the robotization and industrialization of livestock farming, and decrease human attention for the animals in these systems (Krampe et al. 2021). However, the veterinarians also identified ways to mitigate the distance between the agricultural sector and society: the interest of part of society in animal welfare seems to be a starting point. PLF technologies could play a role in this if they are used to improve animal welfare. With this strategy, two main problems may arise in practice. First, PLF is not unilaterally beneficial for animal welfare, but also involves potential threats (Tuyttens et al. 2022). Particularly the more indirect harms, such as technology-mediated effects on the moral status of farm animals in society, are difficult to predict and prevent (Tuyttens et al. 2022). Second, the success of PLF in reconnecting agriculture and society may be dependent on the definition of animal welfare it adopts. The sector may incorporate a health- and production-centered concept of animal welfare into PLF, whereas citizens may expect a concept which does justice to the animals’ subjective experiences and emotions (Dawkins 2021). The veterinarians also suggested that the sector could reconcile society by presenting facts that show that PLF technologies decrease common problems in pig production, such as cannibalism and antimicrobial use. However, convincing people through facts may be difficult as misconceptions on and prejudices about livestock farming often persist (Sonntag et al. 2019). There may be a chance in “explaining and discussing with people” as one veterinarian put it. The format in which this could be realized on a large scale in practice has yet to be explored.

## Conclusion

The aim of this study is to make sense of how veterinarians perceive the use of smart sensing technologies in the context of public concerns related to current pig production in Europe. With this we close the empirical gap and open the way for a comprised socio-ethical analysis of PLF in the context of pig production including the perceptions of various key stakeholders. Based on the analysis of the interviews, we draw the following main conclusions. (1) Veterinarians agree on broad concepts, such as their advisory role and the supporting role of PLF technologies. What these concepts entail in detail, however, varies within the group of pig veterinarians. (2) The veterinarians’ elaborations on relationships among different stakeholders (including themselves) show that they share positions with different groups of society. They are aware of and reflect on competing interests of these groups. (3) Some veterinarians include the development and provision of PLF tools in their advisory activities. This demonstrates that they play an active role in the emerging field of sensor technologies in pig farming. (4) The veterinarians also have a clear idea of the chances and challenges PLF may pose. The use of PLF technologies in pig husbandry as a means to mitigate public concerns must not ignore the present distance between agriculture and society. According to the veterinarians, PLF should not reinforce the misconceptions resulting from this distance. Applying PLF to improve pig welfare, a topic in which part of society is interested, is seen as a starting point to reconcile the sector and the public. These conclusions indicate the availability of building blocks that enable veterinarians to play a mediating role between different stakeholders and emerging sensor technologies. We define these deliberately as building blocks, because they enable rather than automatically lead to the mediating role of veterinarians. Such a process of role taking is also influenced by external obstacles, such as financial dependencies, that may prevent veterinarians to fulfill this role in practice. Future research should investigate which incentives could be offered to veterinarians so that they make use of their potential and take a proactive stance in the public debate on pig production. Further research is also needed to determine whether the concept of human animal care may be common ground among all stakeholders involved. If so, it may be essential to implement this concept into PLF technologies in order to address societal concerns related to pig production.

## Electronic supplementary material

Supplementary Material 1

Supplementary Material 2
