# Supplementary material for: As if you were hiring a new employee: on pig veterinarians’ perceptions of professional roles and relationships in the context of smart sensing technologies in pig husbandry in the Netherlands and Germany
Source: Agric Human Values. 2023 May 1:1–14. Online ahead of print. doi: 10.1007/s10460-023-10450-6 (PMC10150679; doi:10.1007/s10460-023-10450-6)
Supplement: Supplementary file 1 — Supplementary Material 1 [file 10460_2023_10450_MOESM1_ESM.docx]

**As if you were hiring a new employee: on pig veterinarians’ perceptions of professional roles and relationships in the context of smart sensing technologies in pig husbandry in the Netherlands and Germany**

Online Resource 1: COREQ checklist – criteria for reporting qualitative studies (Tong et al. 2007)

Agriculture and Human Values

Mona F. Giersberg* and Franck L. B. Meijboom

Animals in Science and Society, Department Population Health Sciences, Faculty of Veterinary Medicine, Utrecht University, Utrecht, Netherlands

*Correspondence: m.f.giersberg@uu.nl

Table 1: COREQ checklist – criteria for reporting qualitative studies (adapted from Tong et al. 2007)

| **No Item** | **Guide questions/descriptions** | **Title article** |
| --- | --- | --- |
| Domain 1: research team and reflexivity | | |
| Personal characteristics | | |
| 1. Interviewer | Which author conducted the interviews? | The first author (MFG) conducted the interviews. |
| 2. Credentials | What were the researcher’s credentials? | PhD |
| 3. Occupation | What was their occupation at the time of the study? | MFG was employed as researcher animal welfare/sustainable animal stewardship at Utrecht University. |
| 4. Gender | Was the researcher male or female? | MFG is female. |
| 5. Experience and training | What experience or training did the researcher have? | MFG had > 8 years of experience in quantitative research, and > 3 years in non-empirical research. This is her first qualitative study. |
| Relationship with participants | | |
| 6. Relationship established | Was a relationship established prior to study commencement? | MFG did not know the participants personally and had not worked with them before. |
| 7. Participant knowledge of the interviewer | What did the participants know about the researcher? | Participants knew that MFG is a researcher affiliated with Utrecht University interested in farm animal welfare and the socio-ethical aspects around this topic. |
| 8. Interviewer characteristics | What characteristics were reported about the interviewer? | Participants were told that MFG was trained as a veterinarian in Germany but has always been working in research. |
| Domain 2: study design | | |
| Theoretical framework | | |
| 9. Methodological orientation and theory | What methodological orientation was stated to underpin the study? | Reflexive thematic analysis (TA) within a critical realist paradigm. |
| Participant selection | | |
| 10. Sampling | How were participants selected? | Purposive/convenience? |
| 11. Method of approach | How were participants approached? | Participants were invited via e-mail to take part in the study. |
| 12. Sample size | How many participants were in the study? | 12 pig veterinarians participated in the study. |
| 13. Non-participation | How many people refused to participate or dropped out? Reasons? | 10 veterinarians did not respond to the e-mail invitation; 2 declined participation because of retirement; 1 declined participation because of time constraints. |
| Setting |  |  |
| 14. Setting of data collection | Where was the data collected? | Data collection took place online, via MS Teams; participants joined either from home or their office. |
| 15. Presence of non-participants | Was anyone else present besides the participants and researchers? | No. |
| 16. Description of sample | What are the important characteristics of the sample? | 7 pig veterinarians from NL: 1 female, 6 males, 3-35 years of work experience  5 pig veterinarians from DE: 1 female, 4 male, 6-31 years of work experience |
| Data collection | | |
| 17. Interview guide | Were questions, prompts, guides provided by the authors? Was it pilot tested? | The interview guide is published as Online Resource 2. It was tested with colleagues as the sample was too small to recruit potential participants for piloting. |
| 18. Repeat interviews | Were repeat interviews carried out? If yes, how many? | No. |
| 19. Audio/visual recording | Did the research use audio or visual recording to collect the data? | 11 interviews were video- and audio recorded. In 1 case consent for recording was not given. In this case, consent was given to take notes during the interview. |
| 20. Field notes | Were field notes made during and/or after the interview? | During the 1 interview that was not recorded, notes were taken almost verbatim. |
| 21. Duration | What was the duration of the interviews? | The duration of the interviews ranged between 27 and 47 min. |
| 22. Data saturation | Was data saturation discussed? | No, the reflexive TA approach does not make use of data saturation. |
| 23. Transcripts returned | Were transcripts returned to participants for comment and/or correction? | No. |
| Domain 3: analysis and findings | | |
| Data analysis | | |
| 24. Number of data coders | How many data coders coded the data? | MFG coded all data. |
| 25. Description of the coding tree | Did authors provide a description of the coding tree? | No. |
| 26. Derivation of themes | Were themes identified in advance or derived from the data? | In line with the reflexive TA approach, themes were derived from the data. |
| 27. Software | What software, if applicable, was used to manage the data? | Data were analyzed and managed with NVivo version 12 pro. |
| 28. Participant checking | Did participants provide feedback on the findings? | No. |
| Reporting | | |
| 29. Quotations presented | Were participant quotations presented to illustrate the themes/ findings? Was each quotation identified? | Quotations of all participants were presented; each of them was identified and pseudonymized (TA1-5, DA1-7) |
| 30. Data and findings consistent | Was there consistency between the data presented and the findings? | Yes. |
| 31. Clarity of major themes | Were major themes clearly presented in the findings? | Yes, there are four major themes, named and summarized at the beginning of the results section. |
| 32. Clarity of minor themes | Is there a description of diverse cases or discussion of minor themes? | The first two major themes include two minor themes each. These are indicated by small letters (a,b). |

**Reference**

Tong, Allison, Peter Sainsbury, and Jonathan Craig. 2007. Consolidated criteria for reporting qualitative research (COREQ): A 32-item checklist for interviews and focus groups. *International Journal for Quality in Health Care* 19: 349–357. https://doi.org/10.1093/intqhc/mzm042.
