# Supplementary material for: As if you were hiring a new employee: on pig veterinarians’ perceptions of professional roles and relationships in the context of smart sensing technologies in pig husbandry in the Netherlands and Germany
Source: Agric Human Values. 2023 May 1:1–14. Online ahead of print. doi: 10.1007/s10460-023-10450-6 (PMC10150679; doi:10.1007/s10460-023-10450-6)
Supplement: Supplementary file 2 — Supplementary Material 2 [file 10460_2023_10450_MOESM2_ESM.docx]

**As if you were hiring a new employee: on pig veterinarians’ perceptions of professional roles and relationships in the context of smart sensing technologies in pig husbandry in the Netherlands and Germany**

Online Resource 2: Interview protocol matrix and interview guide

Agriculture and Human Values

Mona F. Giersberg* and Franck L. B. Meijboom

Animals in Science and Society, Department Population Health Sciences, Faculty of Veterinary Medicine, Utrecht University, Utrecht, Netherlands

*Correspondence: m.f.giersberg@uu.nl

**Overall research question (RQ):**

How do veterinarians evaluate the current and potential role of PLF (precision livestock farming) in addressing societal concerns over pig production?

**Sub-RQs:**

RQ #1: How do veterinarians evaluate current pig production and possible societal concerns in general?

RQ #2: How do veterinarians evaluate PLF?

RQ #3: To which extent and how do veterinarians indicate the potential of PLF as an answer to societal criticism of pig production?

RQ #4: To which extent and how do veterinarians indicate the potential of PLF to address the diversity of concerns?

RQ #5: To which extent and how do veterinarians indicate the potential of PLF to bring citizens and farmers and their visions of pig husbandry closer together?

Table 1: Overview of the interview protocol matrix – aligning research questions (RQ) with interview questions (IQ) (Castillo-Montoya 2016, modified)

|  | **Background information** | **RQ #1** | **RQ #2** | **RQ #3** | **RQ #4** | **RQ # 5** |
| --- | --- | --- | --- | --- | --- | --- |
| **IQ #0** | X |  |  |  |  |  |
| **IQ #1** | X |  |  |  |  |  |
| **IQ #2** | X |  |  |  |  |  |
| **IQ #3** | X | (X) |  |  |  |  |
| **IQ #4** |  | X |  |  |  |  |
| **IQ #5** |  | X |  |  |  |  |
| **IQ #6** |  | X |  |  |  |  |
| **IQ #7** |  |  | X |  |  |  |
| **IQ #8** |  |  | X | (X) | (X) |  |
| **IQ #9** |  |  |  |  |  | X |
| **IQ #10** |  |  |  |  |  | X |
| **IQ #11** |  |  |  | X | X |  |
| **IQ #12** |  |  |  | X |  |  |
| **IQ #13** |  |  |  | X | X | X |
| **IQ #14** |  |  | X |  |  |  |
| **IQ #15** |  |  | X | X | X | X |
| **IQ #16** | X | X | X | X | X | X |

**Interview guide**

Script prior to the interview:

- *Explain the aim of the research project (develop and implement an innovative pig production system that improves pig welfare and resilience, provides the industry with smart sensing tools to prevent problems, allows for the production of pork in a sustainable way that is accepted by society.*
- *Explain the topic of the interview (role of PLF, definition: precision livestock farming, management of livestock using the principles and technologies of process engineering; relies on automatic monitoring of animals an related environmental processes by smart sensing technologies; suitable processes e.g.: monitoring of animal growth, aspects of animal behavior, thermal micro-environment); degree of integration (single technology vs. ‘digital farm’) and degree of automation (individual feeding stations vs. decision making tools/apps) vary).*
- *Indicate duration of the interview*
- [Review consent form]
- *Permission to video-/ audio record the conversation?*
- *Any questions before beginning of the interview?*

**Working as a veterinarian**

*To begin this interview, I would like to ask you some questions about your background and your work as a veterinarian.*

**IQ #0** With which gender do you identify? How old are you?

**IQ #1** Since when have you been working as a veterinarian?

**IQ #2** With which animals do you mainly work with?

Follow-up: since when have you been working with [animals]?

**IQ #3** How would you describe the clinic/practice/institution you are currently working in?

How would you describe your function and your main tasks within [clinic/practice /institution]?

Follow up: Where is [clinic/practice /institution] located?

**Current and future pig production**

*Thank you for sharing this information. I would like to now ask you a few questions about your experiences regarding pig production.*

**IQ #4** Can you name two to three issues you see with current pig production?

Follow-up: Can you tell me more about what makes you consider these aspects as issues?

[If animal-based issues are mentioned, move to **IQ #5**; if not:]

Can you name two to three animal-based issues you see with current pig production?

Follow-up: Can you tell me more about what makes you consider these aspects as issues?

**IQ #5** Which concerns may be raised by society regarding current pig production?

**IQ #6** What will pig production look like in the future?

Follow-up: Which aspects of this vision do you welcome? Does any of the aspects you mentioned worry you?

**PLF – incident; implications for animals and humans**

[If PLF was mentioned in **IQ #6** refer to it as a transition to **IQ #7**; if not: *PLF technologies which allow for automated animal monitoring are becoming more and more common on commercial pig farms*. If necessary, provide a broad definition: *PLF is the management of livestock by means of the principles and technologies of process engineering. It relies on automatic monitoring of animals and related environmental processes by various smart sensing technologies.*]

**IQ #7** Can you describe a past event on one of your clients’ farms where you came into contact with PLF?

[If never came into contact with PLF on a farm: Can you describe a PLF system in pig production that you read about or that you heard about from a colleague/at a conference?]

What kind of system is it? How does it work?

Do you welcome any of the aspects of this system? Does any of them worry you?

**IQ #8** How does/will the application of PLF affect the animals?

**IQ #9** Which aspects of PLF may be appreciated by farmers?

Follow-up: How did you come to see that farmers would appreciate [aspect(s)]?

**IQ #10** Which aspects of PLF may cause concerns of farmers?

Follow-up: How did you come to see that farmers would be concerned about [aspect(s)]?

**IQ #11** Which aspects of PLF may be appreciated by society?

Follow-up: How did you come to see that society would appreciate [aspect(s)]?

**IQ #12** Which aspects of PLF may cause concerns in society?

Follow-up: How did you come to see that society would be concerned about [aspect(s)]?

**PLF - answer to societal concerns**

*Thank you for your responses. Going back to the beginning of our talk, you mentioned that society is concerned about [aspect(s)] in current pig production.*

**IQ #13** Do you think PLF can be a means to tackle these concerns?

[If yes:] Which of the concerns can be tackled by means of PFL? How should PLF be applied to achieve this?

[If no:] Which characteristics of PLF or its application must be changed so that it can serve as a means to tackle these concerns?

**IQ #14** How would you describe your role as a veterinarian in this process?

**Overall evaluation, closing**

*Taking together the different aspects we have discussed with regard to pig production and PLF,*

**IQ #15** Which aspects of PLF do you rate most important to be pursued in future research?

Which aspects are most important to consider when applying PLF on-farm?

**IQ #16** Before we conclude this interview, is there something regarding pig husbandry or PLF that we have not yet had a chance to discuss?

**References**

Castillo-Montoya, Milagros. 2016. Preparing for interview research: The interview protocol refinement framework. *Qualitative Report* 21: 811–831.
